# Lecture series on gender medicine at Innsbruck and Vienna medical universities: a teaching format in comparison

**DOI:** 10.3205/zma001313

**Published:** 2020-03-16

**Authors:** Sandra Steinböck, Ulrike Nachtschatt, Margarethe Hochleitner

**Affiliations:** 1 Medical University of Vienna, Office for Gender Mainstreaming und Diversity, Vienna, Austria; 2 Medical University of Vienna, Coordination Office for Equal Treatment, Affirmative Action for Women and Diversity, Vienna, Austria; 3 Medical University of Innsbruck, Innsbruck, Austria

**Keywords:** gender medicine, medical education, medical curricula

## Abstract

**Introduction: **Diversity-specific differences in health, illness and access to a health system have meanwhile been studied well. Educating medical students offers good leverage for broadening this knowledge within the medical professions. One approach is to use elective subjects.

**Project Description: **The goal of this work is to compare the lecture series on Gender Medicine at the Medical Universities of Innsbruck and Vienna.

**Results: **The cornerstones of both of these lecture series (topics that vary per semester, various lecturers speaking on a predefined cross-cutting topic) are similar. Various approaches prevail for the target groups and the lecturers as well as the question of credit for external students. Both universities tackle different medical disciplines each semester while concentrating on gender-specific questions.

Teacher evaluation in Innsbruck as well as the feedback from the students in Vienna show that the lecture series have a positive impact on how the various diversity categories influence health and illness.

**Discussion: **Ensuring that a particular leitmotif runs through the various lectures of a lecture series entails increased planning and organizational work. On the other hand, various medical disciplines and their perspectives can be presented in a lecture series.

**Conclusion: **The lecture series are embedded in overall strategies at the two universities. Ensuring the sustainability of the integration of Gender Medicine as a cross-cutting topic in medical education is, however, only possible when combined with other efforts.

## Zusammenfassung

**Einleitung: **Diversitätsspezifische Unterschiede in Gesundheit, Krankheit und Zugang zum Gesundheitssystem sind mittlerweile gut untersucht. Um dieses Wissen innerhalb der medizinischen Berufsgruppen zu verbreitern, ist die Ausbildung der Medizinstudent*innen ein wichtiger Hebel. Ein Ansatzpunkt ist die Nutzung von Wahlfächern.

**Projektbeschreibung: **Ziel ist die vergleichende Darstellung der Ringvorlesungen zu Gender Medizin an den Medizinischen Universitäten Innsbruck und Wien.

**Ergebnisse: **Die Grundpfeiler der Lehrveranstaltung (semesterweise wechselnde Themen, verschiedene Vortragende zu einem vorab definierten, quer zu den Disziplinen liegenden Thema) sind ähnlich. Sowohl bei Zielgruppen und Vortragenden als auch bei der Frage der Anrechnung für Externe gibt es verschiedene Zugänge. Inhaltlich bearbeiten beide semesterweise wechselnde medizinische Fachgebiete mit dem Schwerpunkt auf geschlechtsspezifischen Fragestellungen.

Sowohl die in Innsbruck vorliegende Evaluation durch Lehrende als auch das Feedback der Studierenden in Wien zeigen, dass die Ringvorlesungen die Auseinandersetzung mit dem Einfluss verschiedener Diversitätskategorien auf Gesundheit und Krankheit positiv beeinflussen.

**Diskussion: **Die Sicherstellung eines ‚roten-Fadens‘ in den unterschiedlichen Vorträgen einer zusammenhängenden Ringvorlesung ist mit einem erhöhten Planungs- und Organisationsaufwand verbunden. Dafür können in einer Ringvorlesung verschiedene Fachdisziplinen und ihre Perspektiven zu Wort kommen.

**Schlussfolgerung: **Die Ringvorlesungen sind eingebettet in Gesamtstrategien an den jeweiligen Universitäten. Eine nachhaltige Integration von Gender Medizin als Querschnittsthema der Lehre ist jedoch nur gemeinsam mit anderen Maßnahmen erfolgsversprechend.

## 1. Introduction

Gender Medicine is a relatively young medical discipline. It includes the effects of biological factors (height, weight, hormones, percentage of body fat, muscle mass) and sociocultural factors (education, financial resources, religion, ethnic background or sexual orientation) on health and illness. In addition to women and men, Gender Medicine also includes LGBT persons [1] as well as persons of the third sex. In addition to “age” the category “gender” counts among the most important diversity categories in medicine [2]. Possible gender-specific differences pervade the entire course of medical diagnostics and treatment. Moreover, women and men access the health system differently. Since the turn of the century various universities in Europe and North America have successively built up their Gender Medicine course offerings and attempted to anchor the contents in various forms at their universities. Currently there is, however, no standardized way of going about this. Instead there is a broad range of various methods and steps that can be taken. These include presenting the contents in courses, workshops, guidelines, online courses, public lectures, all the way to structurally anchoring such offerings at the universities [3], [4], [5], [6]. Gender Medicine is, on the one hand, its own discipline. On the other hand, it is a cross-cutting medical topic that is relevant for all medical disciplines with only few exceptions. The discussion of which methods and formats are suitable for presenting Gender Medicine content in a stringent manner has not yet been exhausted [7]. The work at hand thus focuses on a university teaching format, the lecture series, and uses the Medical Universities of Innsbruck and Vienna to show which thoroughly different Gender Medicine teaching goals can be successfully implemented with this format.

## 2. Project Description

The goal of this work is to compare the lecture series on Gender Medicine at the Medical Universities of Innsbruck and Vienna. In Innsbruck since 2006 and in Vienna since 2004 the medical universities have offered a Gender Medicine lecture series for (not only) medical students.

In both Innsbruck and Vienna a wide range of steps are taken to ensure that the category “gender” is sustainably and intersectionally integrated into teaching [6]. At the Medical University of Innsbruck Gender Medicine has been integrated into the compulsory courses for all study programs. These compulsory courses cover Gender Medicine as its own discipline and impart basic principles, definitions, methodological competence or Gender Medicine & Diversity in scientific research and teaching. Parallel to the courses there are guidelines for science and teaching, as well as guidelines for how to give consideration to Gender Medicine questions in diploma theses and doctoral theses, as well as workshops for young scientists and teachers.

At the Medical University of Vienna Gender Medicine is covered in the compulsory courses in the diploma study programs. For teachers there are (electronically available) tools and support offerings, that facilitate the integration of “gender” into the medical university’s courses. Moreover, these tools and support offerings are embedded in strategically anchored measures such as the development of learning objectives and raising teachers’ awareness for the subject through medical teaching offerings.

The format of the ring lecture series permits individual lectures on a particular subject to be drawn together using the lecture contents, which can be at quite a right angle to traditional disciplines.

Precisely “gender” is a subject often dealt with outside the traditional medical disciplines and with an interdisciplinary approach that often also shows the thematic relevance of subjects complementary to medicine, such as, for example, psychology, sociology, law or nursing. In addition, in this way “gender” can be dealt with in its efficacy on both a biological level as well as a sociocultural level in the context of other diversity categories. The format of the lecture series is thus very well suited for Gender Medicine as a cross-cutting subject and the complexity of Gender Medicine issues.

In the following the two lecture series offerings will be compared. In this way various differences, for example in target group or content structure, will be shown, commonalities presented and the two concepts will be described and analyzed in their organizational framework and with their goals. In order to better compare them and for reasons of clarity the most important framework conditions and benchmarks will be presented in a table. In a second step the feedback received from teachers (Innsbruck) and students (Vienna) will be looked at. Both of these studies were conducted several years ago for different reasons and the already available data were employed here. To this extent the results are not comparable. These data do, however, show how the lecture series were greeted and what effects they have and are thus included in the description. Following the results is a discussion of the advantages and disadvantages as well as the conclusions drawn from the experience made when drawing up and implementing the lecture series.

## 3. Results

Although both universities chose the same teaching format, the lecture series, a first look makes clear that both universities work with similarly conceived elements that are directly related to each other, and in other areas with very different approaches.

### 3.1. Formal Concept

The conceptual make-up of the two lecture series is very similar. In the framework of the electives available to students different topics are presented each semester or year. An elective gives students the opportunity to acquire more detailed knowledge in a specific field according to their interests. Even though it is necessary to integrate Gender Medicine into the compulsory medical courses because their content is important, an elective provides especially interested students with an additional opportunity to take a detailed look at a subject. At both medical universities, Innsbruck and Vienna, Gender Medicine as a topic is firmly established in the compulsory courses and in an elective.

The advantage of the lecture series is the broad range of the different contents that are presented. The challenge is to ensure that the lectures form a cohesive overall picture of the subject. This is ensured by setting a clear goal for the contents at both universities: after drawing up a concept for the lecture series content, each lecture is discussed with the person who will present it. Moreover, the first and last lectures in the series serve to bookend the series by commenting on the overall concept and the various lectures and how they mesh with each other. Furthermore, these lectures will serve to present basic Gender Medicine content.

The benchmarks to the particular lecture series are given in table 1 (Tab. 1).

At the Medical University of Innsbruck the lecture series focuses on conveying knowledge not only to the students but also to a much broader audience. The lecture series is conceived for a very broad target group consisting not only of students from other disciplines, but also physicians, members of allied health professions and the interested public. This focus on the broadest possible audience, that is accompanied by advertising and the lack of a final exam, brings in a large audience. On average, 165 persons attend the lecture series in Innsbruck each semester.

At the Medical University of Vienna the target group is defined to be clearly smaller – the lecture series is aimed mainly at university students and primarily those at the Medical University of Vienna. On average 15-20 students attend one of the two lectures series that are offered each semester. The focus is on imparting Gender Medicine knowledge to a group of students who are particularly interested in this subject. These students receive in-depth knowledge in a setting that permits a personal exchange and discussion with the teachers.

The emphasis on diversity and the outreach to other medically relevant categories is more explicit. Thus, once a year there is a diversity lecture series; it puts a huge focus on the other categories of diversity. The discussions on „gender“ and the diversity categories can thus be pursued in an explicit way, raised to a meta-level and form part of the course examination.

#### 3.2. Design of Content

At both medical universities planning for the lecture series starts by drawing up a basic concept for content. Every semester this concept is given to all the teachers at both universities and is discussed with them in a personal talk. At both universities the lecture series starts with an introductory lecture held by the teacher responsible for the lecture series. The introductory lecture presents basic concepts of Gender Medicine as well as the series of lectures and their perspective on the semester’s subject.

In content the two lecture series are thoroughly comparable – medical disciplines and topics that change every semester are presented with a focus on possible gender-specific differences. “Gender” is repeatedly mentioned and analyzed in connection with other diversity categories, as far as these are relevant for the particular subject. The goal is for the audience to familiarize themselves with and acquire an understanding of current, evidence-based, gender and Gender Medicine research findings, and in Vienna they should additionally learn to reflect on these findings. Both medical universities strive to achieve a good balance between lecturers from the basic non-clinical subjects and those from the clinical disciplines. Both Innsbruck and Vienna offer lecture series that deal with a subject in more detail and more depth (special lectures) as well as lecture series that offer a broad overview of a subject (overview lectures). Table 2 (Tab. 2) shows the different nuances in content at the two universities.

Another strategy practiced at the Medical University of Innsbruck in order establish Gender Medicine as a cross-cutting topic is to specifically choose the lecturers. For this purpose, the heads of the clinics and research departments, i.e. the highest persons in the hierarchy, are personally invited to hold a lecture. In this way Gender Medicine research findings and teaching concepts are brought to the various medical disciplines at the highest level, which increases the acceptance of this new medical discipline.

#### 3.3. Feedback from Teachers and Students

The questions discussed in this article are not included in the standardized lecture evaluation. The feedback described in the following refers to surveys that above all serve for a final appraisal of the project.

At the Medical University of Innsbruck the teachers’ perspective was in the foreground, because the teachers had been personally invited and because the organizers of the lecture series view the lectures as an opportunity to build up the content competency on Gender Medicine issues among the lecturers and in the various disciplines and thus to use the lecture series to go beyond the defined student goals, namely to raise awareness for Gender Medicine among the teachers.

The Medical University of Vienna obtained the students’ feedback. The questionnaire concentrated mainly on how the students viewed and experienced the lecture series in order to be able to develop the best possible, customized offerings.

The feedback presented here is thus not comparable, but instead covers various perspectives, all of which, however, are necessary if we are to evaluate the lecture series in the framework of its efficacy and the setting in which it is anchored.

##### 3.3.1 The Perspective of the Teachers

At the Medical University of Innsbruck in 2013 a survey was conducted of the persons who had already lectured in the Gender Medicine lecture series. They were asked for their view of Gender Medicine at the Medical University of Innsbruck. Of the lecturers 62 participated (20 women, 42 men), mean age 52.8 years. Of the respondents 80.65% held a management position. The lecturers’ responses were seen to revolve around three main points. For example, the invitation to lecture on the gender-specific topics of their research field was definitely viewed as a challenge and in the early years of the lecture series there was much uncertainty about what the content of such a lecture should be. At the same time some of the lecturers replied that it was a worthwhile reference showing that they had taken a public look at the gender-specific perspective. Of the lecturers 23 stated that their preparations for their lecture and their review of the Gender Medicine literature on their subject had caused them to deal more consciously with the medical relevance of sex- and gender-specific differences, had expanded their knowledge and that their lecture had prompted them to include this topic in their own lectures. Only seven of the respondents stated that dealing with gender-specific differences in their field had not caused any changes or brought about any new incentives. A large portion of the lecturers experienced the lecture series as well-accepted and established.

##### 3.3.2. The Perspective of the Students

At the Medical University of Vienna the lecture series were evaluated by personally interviewing the students who had attended the lecture series in the years 2007 to 2011.

For the interviews a guideline was developed that contained questions on “Concept and Execution of the Lecture Series,” “Organization of the Lecture Series,” “gender-specific Content in a Medical Degree Program” and “Information on the Respondent.”

The students, a total of approx. 200, were invited by email by the Office for Gender Mainstreaming to participate in the interviews for the purpose of evaluating the lecture series. Eleven students (four female, seven male) agreed to be interviewed. This very small number of voluntary respondents results from the framework conditions for the survey: some of the students invited to the interview were no longer available for reasons that had to do with their current life situation or stated that they were not interested in participating. The very small number of interviews conducted naturally means that the results are not representative.

All 11 persons interviewed gave an overall positive evaluation of the lecture series organized by the Office for Gender Mainstreaming. They described the lecture series as a “great idea,” “exciting” and “very good.” They especially praised the wide range of topics presented. All of the interviewed students also stated that they were satisfied to very satisfied with the interdisciplinary concept of the lecture series.

As the reasons for attending the lecture series, five of the interviewed students stated that they had a personal interest in the topic of “gender.” Six of the interviewed students stated that they had decided to attend for practical reasons, for example in order to have the required number of elective hours for that semester. In the course of attending the lecture series some of the interviewed students developed an interest in the subject of Gender Medicine.

When asked how they had profited from attending the lecture series, a large portion of the interviewed students stated their perspective had broadened and that because of the lecture series they now took a more differentiated view of medical questions.

## 4. Discussion

Meanwhile there is a broad consensus of opinion that “gender” is an important influencing factor on health and health inequalities [1]. An important and recognized leverage for the distribution of knowledge within the medical professions is, as also recommended by the WHO [10], medical education. For this reason, the Medical Universities of Innsbruck and Vienna employ a number of strategies and activities to ensure that their graduates have acquired gender-specific knowledge.

One step on the way to sustainable and comprehensive integration of the category “gender” in medical education can be the specific course format of a lecture series. Experience has shown that this possibility for thematization of gender-specific aspects in medicine is a possibility often chosen in this framework [7]. A possible reason for this is that teachers who are convinced of the relevance of Gender Medicine are addressed and feel addressed to hold a lecture, which keeps resistance to a minimum. Moreover, it is a good opportunity to make public the Gender Medicine competence at the particular medical university, even if it was previously not explicitly known as “gender.” Thus, the organizers work with resources already available at the particular medical university and put these resources to best use. The lecture series as a format can be easily adapted to venue-specific prerequisites. Different goals, the integration of already available competences and experts or the use of briefly opening windows of opportunity for the purpose of structurally anchoring Gender Medicine offerings are thus easier to implement.

At the Medical Universities of Vienna and Innsbruck the lecture series for gender-specific medical topics are set up very well; they are well established and are very well accepted by the teachers and students. On the one hand, this is shown by the formally obtained feedback (for teachers, the Medical University of Innsbruck; for students, the Medical University of Vienna) as well as the informal feedback received by the teachers responsible for the lecture series. The common ground enjoyed by the formats developed by the two medical universities includes the joint work-up of a subject by various experts, each of whom contributes their own competence and perspective. The broad range of the lecturers’ specialization in the field of medicine and adjacent professions is one factor in making it possible for Gender Medicine expertise at the two universities to be built up and to expand. The character of “gender” and Gender Medicine as interdisciplinary cross-cutting subjects is thus very much taken into account.

While Innsbruck with its very broadly defined target group achieves broad public awareness, Vienna with its small number of participants and the possibility to join in discussions with the teachers provides a more detailed, in-depth lecture and an intense look at the lecture subject. The broad spectrum of the audience in Innsbruck entails the risk of being able to go into less detail. On the other hand, the intense work with the subject practiced at the Medical University of Vienna is at the expense of achieving a broader impact.

It must be emphasized that the lecture series demands more time and effort for planning and organization, also with a view to the everyday organizational responsibilities such as checking attendance, performing evaluation or coordinating schedules. What the person in charge of the concept for the lecture series must ensure is that the lectures do not wind up being a number of random, divergent lectures, but instead a series of lectures with a visible common thematic thread for students and teachers. This demands considerable time and energy as well as an open discussion process with the lecturers when working out the fine details of the lecture series.

In the Literature the integration of Gender Medicine in the compulsory curriculum is seen as the most sustainable and effective anchoring strategy [5]. In this way it is ensured that all students are confronted with the topic. Integration into the compulsory curriculum is a long-range job, demands great assertiveness and adequate resources, as the experience obtained when revising the curriculum at Charité in Berlin shows [3], [4]. By comparison, anchoring an elective is much easier, but also entails the disadvantage of achieving less public awareness and not being as sustainable.

## 5. Conclusion

The lecture series format is good for presenting the cross-cutting topic of Gender Medicine and can be adapted to meet various goals. The particular strengths and weakness of the two models are seen in detail only against the background of the overall concept for the integration of Gender Medicine in the medical curriculum of the particular university. The two approaches presented here can be successful – and the goals staked out for the two venues are also met in the examples discussed here. However, one format alone, no matter how it is designed, is not able to ensure sustainable integration of Gender Medicine as a cross-cutting topic in medical education. This makes it necessary to combine the structural level of curriculum development and the elaboration of teaching goals with course offerings for students as well as with corresponding measures to raise awareness among teachers.

## Competing interests

The authors declare that they have no competing interests. 

## Figures and Tables

**Table 1:**
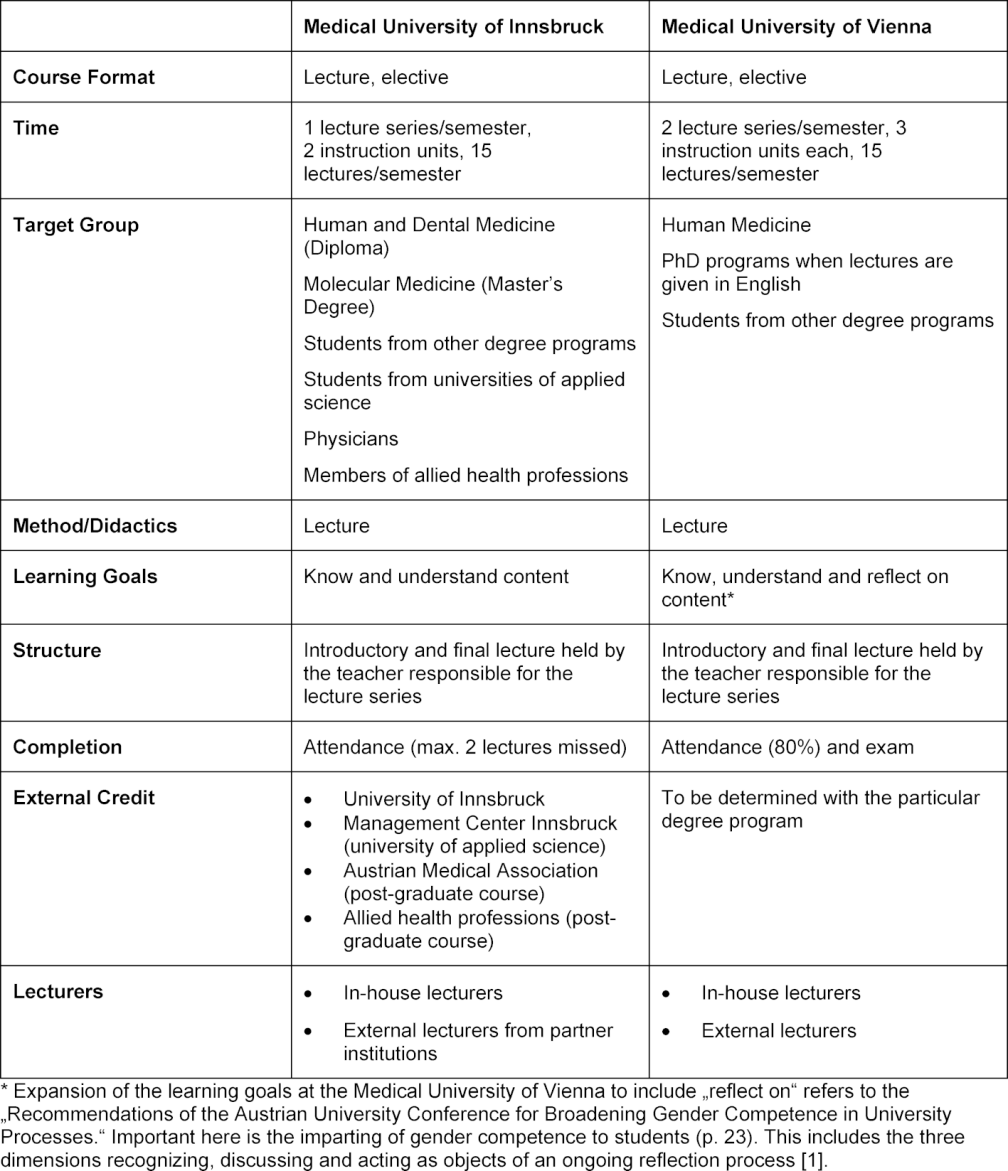
Benchmarks to the particular lecture series

**Table 2:**
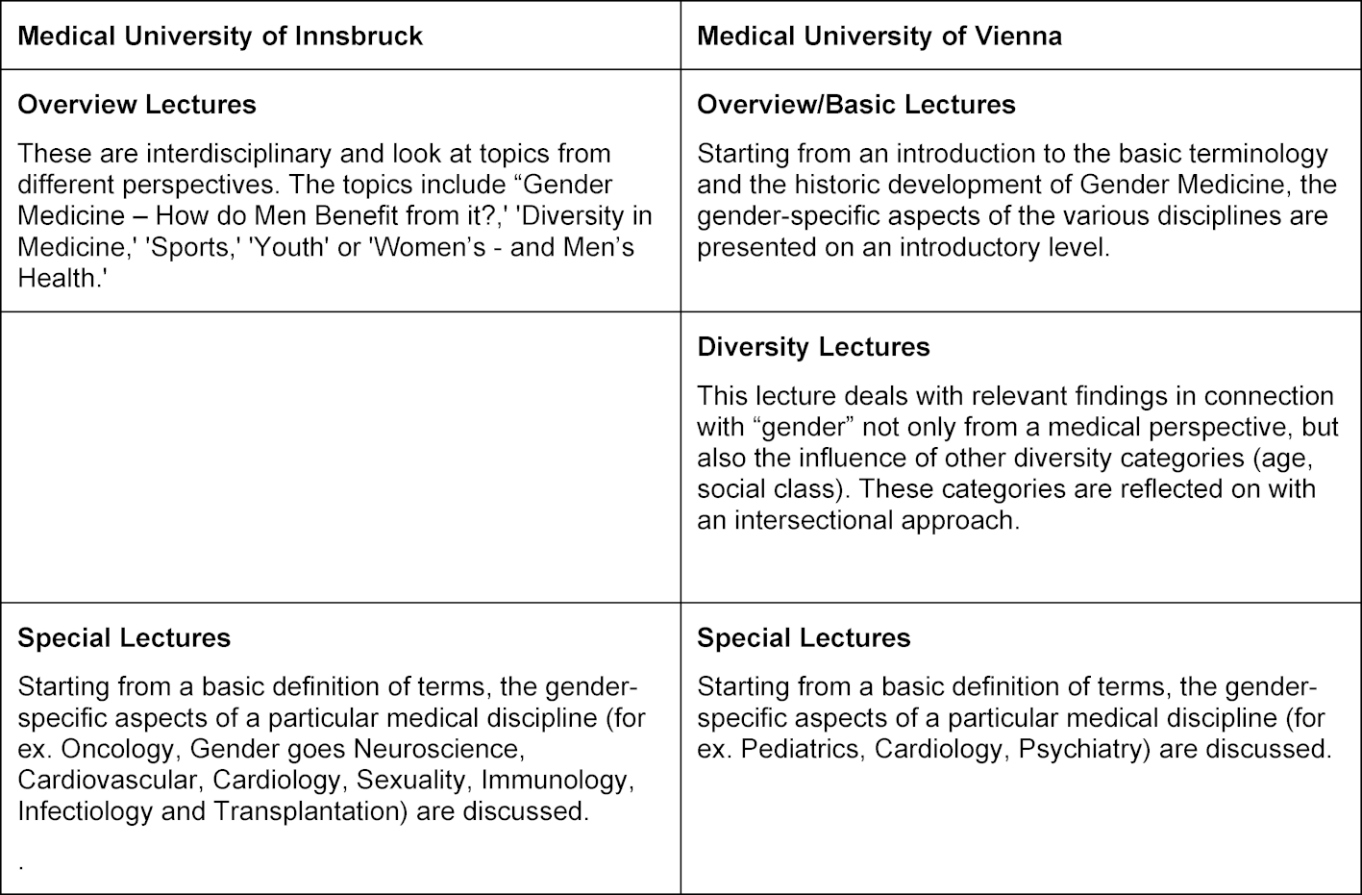
Topics of the lecture series
